# Feral Cat Populations and Feline Retrovirus Prevalence in San Mateo County, California in Three Time Periods between 2001 and 2016

**DOI:** 10.3390/ani12243477

**Published:** 2022-12-09

**Authors:** Charlotte H. Edinboro

**Affiliations:** Exponent, Inc. Health Sciences, Menlo Park, CA 94025, USA; cedinboro@exponent.com

**Keywords:** feral cats, shelter population trends, FeLV prevalence, FIV prevalence

## Abstract

**Simple Summary:**

The prevalence of feline retroviruses in San Mateo County, California was unknown in 2004. Published prevalence in studies worldwide has varied widely, and veterinary guidelines for management of retrovirus-positive cats have not addressed feral cats. This study examined retrovirus prevalence in feral cats presented to a large humane society’s spay/neuter clinic over a 15-year period, in the setting of population trends in the shelter’s total and feral cat admissions, and numbers of cats presented for spay/neuter procedures. The prevalence differed from earlier studies in Florida and North Carolina (higher for one retrovirus and lower for another), even as feral cat and total cat presentations decreased in San Mateo County, suggesting that retrovirus surveillance should continue in this area.

**Abstract:**

This study was initiated in 2004 because the prevalence of feline leukemia virus (FeLV) and feline immunodeficiency virus (FIV) infections in feral cats in San Mateo County (SMC) was not known. The cities attributed to the feral cat population presented to the Peninsula Humane Society & SPCA’s Spay/Neuter Clinic and to the Shelter itself were analyzed to examine potential geographic concentrations of feral cats with positive retroviral status. Trends in FIV and FeLV status were examined in three 3-year periods (2001–2003, 2005–2007, and 2014–2016). Population trends over the 15 years of this study for feral cats admitted to the Shelter were also examined. In each study period, more female feral cats were presented to the S/N Clinic (54.06%, 57.37%, 54.89%). FIV prevalence increased from 5.52% to 6.41% (*p* = 0.29) from the first to third period; FeLV prevalence decreased significantly from 1.73% to 0.29% (*p* = 0.01). Significantly more FIV-positive males than females were identified each year and for each period (*p* < 0.01). The four largest SMC cities were the major source of feral cats to the Shelter, S/N Clinic, and of FIV- and FeLV-positive cats in the first two periods; in the third period, 50% of feral cats to the Shelter and of FeLV-positive cats were from these cities. Despite a 61.63% reduction in feral cat admissions to the S/N Clinic, the FIV prevalence for males remained similar and increased for females. The retrovirus prevalence suggests the need for continued testing and surveillance of FIV among SMC free-living cats.

## 1. Introduction

San Mateo County (SMC), the high socio-economic status area on the peninsula south of the city and county of San Francisco, California, is a mixed suburban and rural county with 20 incorporated towns and cities [1]. In 2000, 2010, and 2016, the human population was reported to be 707,161 [2]; 719,699; and 767,906 [3], respectively. In SMC, as elsewhere, outdoor cats may be owned, stray, or unsocialized (“community cats”); their nature may be not obvious from a distance, and their numbers may be occasionally bothersome.

One method to control the population of outdoor, presumably unsocialized or feral cats is trap-neuter-return or -release (TNR). TNR programs may include testing for feline leukemia virus (FeLV) and feline immunodeficiency virus (FIV), vaccination for upper respiratory viruses (FVRCP) and rabies virus, and microchipping. Ear-tipping has been adopted as a recognized sign to indicate association with established TNR colonies. Cats with ear-tips can be more easily identified, monitored, and released when found in traps without the need to transport them to clinics. TNR programs have been shown to decrease feral cat populations more successfully than wholesale euthanasia and vaccinations reduce the spread of infectious diseases inside and outside colonies and prevent the introduction of new infectious diseases [4,5,6,7,8,9]. De-populating an area can create an empty niche for new free-roaming cats [10,11]. Instead, through feral cat population stabilization, attrition, and removal of socializable kittens and adults, no vacuum is created [12].

Peninsula Humane Society & SPCA (PHS) is a large non-profit open-admission shelter that holds SMC’s animal control contract [13]. PHS has registered feral cat colony caretakers since the early 1990s. On a daily basis, the PHS Spay/Neuter (S/N) Clinic serves its animals before adoption as well as publicly owned animals, including feral cats from individuals and organized feral cat caretaking groups. One of these groups, Homeless Cat Network (HCN), is a loose affiliation of caretakers, started over 30 years ago, and incorporated over 25 years ago [14]. HCN has over 100 actively managed colonies, 300 volunteers, and a Socialization Center for kittens and friendly adult cats [14]. Project Bay Cat (PBC), a collaboration between HCN; PHS; the city of Foster City, California; and the Sequoia Audubon Society, was established in 2004 to address many cats that had apparently been abandoned at the western edge of the San Mateo Bridge [15]. Initially, PBC cared for 175 cats; by 2016, there were 36 cats, and as of 2020, only one cat remained in the area. In total more than 100 kittens and friendly adult cats had been adopted from this colony [15].

FeLV and FIV are feline retroviruses that vary in clinical presentation and clinical outcomes; over the time of this study, these have been studied and better understood, even as research continues [16,17]. Briefly, the transmission of FeLV occurs in cats in close contact with three currently identified outcomes: progressive infection in cats with insufficient immunity; regressive infection with virus contained but not eliminated; and abortive infections, in which only FeLV antibodies remain [17]. Cats with progressive FeLV infections have shorter survival times than those with regressive infections [17]. FIV is transmitted primarily in saliva via bite wounds, as well as by close contact without fighting [17]. Over time, FIV-infected cats may develop chronic infections and inflammatory conditions, cancer, and other immune-deficient conditions [17].

In 1993, SMC created a program [18,19], funded by dog and cat licenses, to make vouchers available for spaying/neutering of owned and feral animals. These vouchers have been used by HCN and other caretakers of feral cat colonies. One stipulation of voucher use for feral cats is that these cats be tested for FeLV and FIV; if they test positive for one of these retroviruses, the cats are humanely euthanized. As of 2002, reported prevalence of FeLV and FIV ranged between 3.7% and 5.8% and between 2.3% and 6.5%, respectively, in studies of feral cats in the southeastern U.S. [20,21]. The results of retroviral testing among feral cats, tested as part of a spay/neuter voucher program in seven sites in Santa Clara County (SCC), immediately south of SMC, yielded a prevalence of 1.6% and 2.6% for FeLV and FIV, respectively, for fiscal year 2003 [22]. These low prevalence numbers, in addition to anecdotal reports and web-based articles [23,24], suggesting that testing kits for FeLV and FIV were insufficiently sensitive or specific and that cats with positive status can continue to live good quality lives, led some members of local feral cat groups to object to the testing and euthanasia requirements associated with the use of the SCC spay/neuter voucher system [25]. The retroviral testing and euthanasia requirements were eliminated in SCC in 2004. Certain feral cat caretakers in SMC, citing the SCC prevalence for FeLV and FIV, also objected to requirements for testing and euthanasia of retrovirus-positive feral cats associated with the SMC spay/neuter vouchers, and ceased using them to pay for S/N Clinic services.

This study was initiated in 2004 as the prevalence of FeLV and FIV infections in feral cats in SMC was not known. To elucidate the retroviral prevalence in feral cats in SMC, the cities attributed to the feral cat population presented to the S/N Clinic and to the Shelter itself were analyzed to examine potential geographic concentrations of feral cats with positive retroviral status. Trends in FIV and FeLV status among the feral cats presented to the S/N Clinic were examined in three 3-year periods (2001–2003, 2005–2007, and 2014–2016). Population trends over the 15 years of this study for feral cats admitted to the Shelter were also examined.

## 2. Materials and Methods

The S/N Clinic maintains paper records for owned cats, dogs, and other owned animals and for feral cats. From these records, information on feral cats for the three 3-year periods, 2001–2003, 2005–2007, and 2014–2016, was entered into a statistical database for analysis (SPSS for Windows Release 11.5.0, IBM SPSS Statistic, version 20, and IBM SPSS Statistics version 25, IBM^®^ SPSS^®^ Statistics, Chicago, IL, USA). Cat information included date of presentation, sex, breed, FeLV and FIV status, color, prior neuter status, presence of tattoos, and medical comments such as pregnancy status and number of fetuses. Caretaker information included initials, zip code and city of residence, and ear-tip preference. Boxes containing the paper records were periodically sent to storage and were not later available to access for this study. Thus, this was a convenience study, based on the records that were available. During the time of this study, the shelter used the SNAP Combo FeLV Ag/FIV Ab test (IDEXX Laboratories, Inc., Westbrook, ME, USA). Between 2001 and 2016, records for all live cats and feral cats presented to the Shelter were provided electronically; for cats identified as feral, information included dates of admission, disposition (return to owner, euthanized, died, adopted), and associated city. The Shelter information was obtained to compare feral cat admissions to the Shelter and to the S/N Clinic over the study periods.

Duplicate identification numbers and record entries for S/N Clinic feral cats were noted and reconciled through inspection, when possible. Because of incomplete records, inconsistencies remained in some of the data; therefore, numbers did not always sum to expected total numbers of cats in various categories. Trends of live cat intake, including feral cats, admitted to the Shelter between 2001 and 2016 and in the three-year periods and to the S/N Clinic were analyzed by the extended Mantel-Haenszel test (χ^2^_MH_) to test for changes in admissions using Epi-Info (Epi Info, version 7, CDC, Atlanta, Georgia) [26]. Prevalence of FeLV and of FIV was calculated for each of these years and for each period; trends were evaluated using χ^2^_MH_. The relationships between FeLV and FIV status and sex were compared using the χ^2^ statistic. Relationships between city of caretaker residents (for the S/N Clinic cats) and city of record (for Shelter cats) were compared for the study periods using the χ^2^ statistic. The temporal trend in feline admissions to the Shelter was evaluated by linear regression, using Excel (Microsoft® Excel 2002 SP3, Microsoft Corporation, Redmond, WA, USA). A *p*-value < 0.05 was considered to be statistically significant.

Between 2006 and 2008, PHS used a mobile van to provide spay/neuter services for dogs and cats in larger cities in SMC and surrounding counties [27]. Feral cats were among the cats served, but were not the focus of the program. It was therefore not possible to determine how many of the total number of cats were feral; these cats were not included in this analysis.

## 3. Results

### 3.1. S/N Clinic

**Figure 1:**
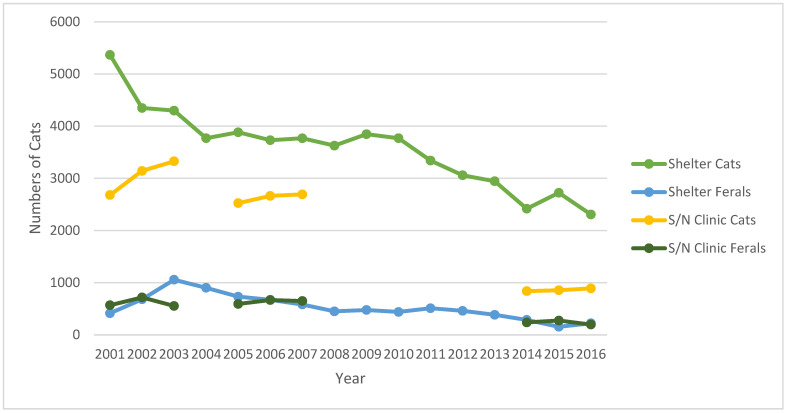
Numbers of cats presented to the PHS Shelter and Spay/Neuter Clinic, 2001–2016.

Overall numbers of cats presented to the S/N Clinic during the three periods of study were 10,989 (2001–2003); 9787 (2005–2007); and 3293 (2014–2016). The feral cats represented 16.82% (1848); 19.60% (1918); and 21.53% (709) of these cats during the three periods, respectively (Table 1, Figure 1).

**Figure 2:**
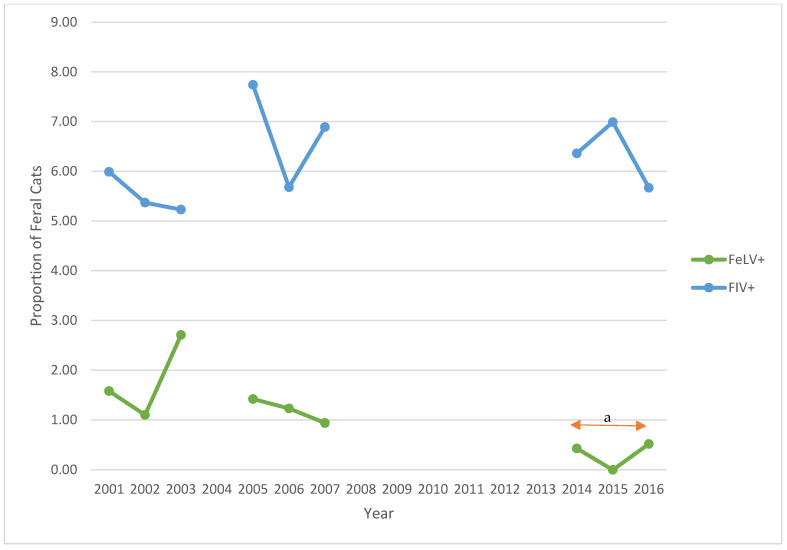
Prevalence of feline leukemia virus-positive (FeLV+) and feline immunodeficiency virus-positive (FIV+) feral cats presented to the PHS Spay/Neuter Clinic, 2001–2003, 2005–2007, and 2014–2016. Note: a—*p* = 0.01 compared with previous two periods.

**Figure 3:**
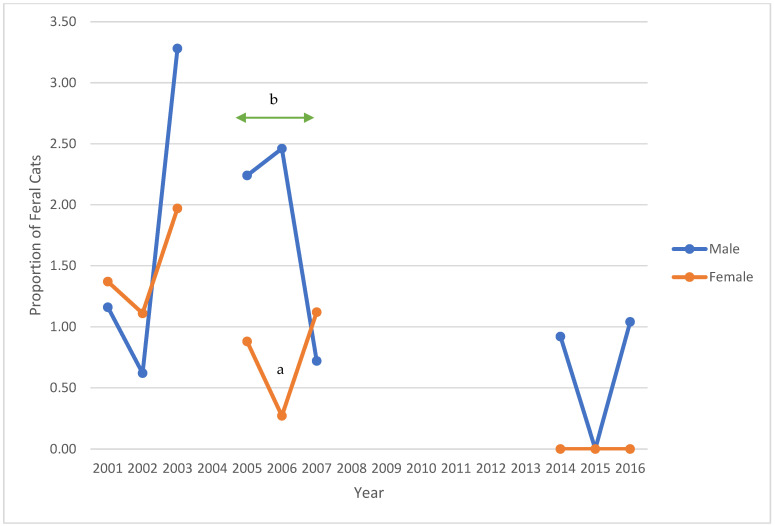
Prevalence of feline leukemia virus-positive feral cats, stratified by sex, presented to the PHS Spay/Neuter Clinic, 2001–2003, 2005–2007, and 2014–2016. Notes: a—*p* = 0.02; b—*p* = 0.04 across the period.

**Figure 4:**
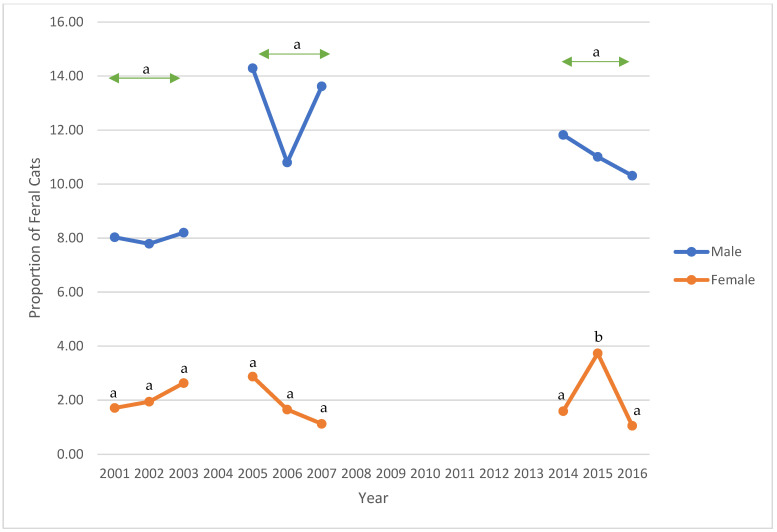
Prevalence of feline immunodeficiency virus-positive feral cats, stratified by sex, presented to the PHS Spay/Neuter Clinic, 2001–2003, 2005–2007, and 2014–2016. Notes: a—*p* <0.01 across each period and for each year (2001, 2002, 2003, 2005, 2006, 2007, 2014, and 2016); b—*p* < 0.02 for 2015.

Between 2001 to 2003, retroviral status was complete for all cats. For those with retroviral data, 32 (1.73%) and 102 (5.52%) feral cats tested positive for FeLV and FIV, respectively (Table 2). The prevalence of FeLV or FIV did not vary significantly across this period (*p* = 0.09 and 0.84, respectively). As 76 of 1848 (4.11%) feral cats had missing information for sex, further analysis was completed for 1772 cats (814 males, 958 females) (Table 3, Figure 2, Figure 3 and Figure 4). Twenty-eight of 1772 (1.58%) cats tested positive for FeLV; 85 (4.80%) tested positive for FIV. FeLV prevalence was not significantly different between male and female cats, while FIV prevalence between males and females differed significantly each year and across the period (*p* < 0.01 each year and across the period). Four cats, all males, tested positive for both retroviruses. Overall, 22 males, 31 females, and one cat of unknown sex had already been altered, and three cats had tattoos indicating prior spay procedure.

Between 2005 and 2007, FeLV and FIV status was missing for 67 (3.49%) and 47 cats (2.45%), respectively. For those with retroviral data, 22 of 1851 (1.19%) and 126 of 1871 (6.73%) feral cats tested positive for FeLV and FIV, respectively (Table 2). No significant differences were noted for FeLV or FIV prevalence across this period (*p* = 0.75 and 0.35, respectively). In this period, 816 males, 1098 females, and four cats of unknown sex were presented to the S/N Clinic (Table 3). FeLV prevalence for females was significantly lower than for males in 2006 (*p* = 0.02). There was a significant difference in FeLV prevalence between males and females in the second period (*p* = 0.04). FIV-positive prevalence significantly differed between males and females (*p* < 0.01 for each year and across the period) (Figure 2, Figure 3 and Figure 4). Between 2005 and 2007, four male and two female cats tested positive for FeLV and FIV. Twenty-four male and 32 female cats had already been altered; five cats had tattoos.

Between 2014 and 2016, FeLV and FIV status was missing for nine and seven cats, respectively. For those with retroviral data, 2 of 700 (0.29%) and 45 of 702 (6.41%) feral cats tested positive for FeLV and FIV, respectively (Table 2). Again, no significant differences were noted for FeLV or FIV prevalence across this period (*p* = 0.52 and 0.85, respectively). In this third period, 318 males, 387 females, and four cats of unknown sex were presented to the S/N Clinic (Table 3). FeLV-positive prevalence among male cats had decreased compared with the previous periods. There were no female FeLV-positive cats. FIV prevalence among male cats was significantly different from female cats (*p* < 0.01 in 2014 and 2016, *p* < 0.02 in 2015; *p* < 0.01 across the period) (Figure 2, Figure 3 and Figure 4). Information for nine feral cats was missing (1.27%). During this third period, no cats tested positive for both FIV and FeLV. Six males, three females, and two cats of unknown sex had already been spayed; no cats were presented with tattoos.

Over the three study periods, the prevalence of FeLV decreased significantly from 1.73% to 0.29% (*p* = 0.01), while the prevalence of FIV increased from 5.52% to 6.41% (*p* = 0.29) (Table 2). In each of the three study periods, more female than male feral cats were presented to S/N Clinic. For each year of the study, and for each period, there were significantly more FIV-positive males than females (*p* < 0.01; *p* < 0.02 for 2015) (Table 3). Despite a reduction of 61.63% in feral cat admissions to the S/N Clinic in the third period, compared with the first period, the prevalence of FIV in the third period for males remained similar across the years 2014–2016 and increased for females in 2015.

### 3.2. Sources of Feral Cats Presented to S/N Clinic

**Figure 5:**
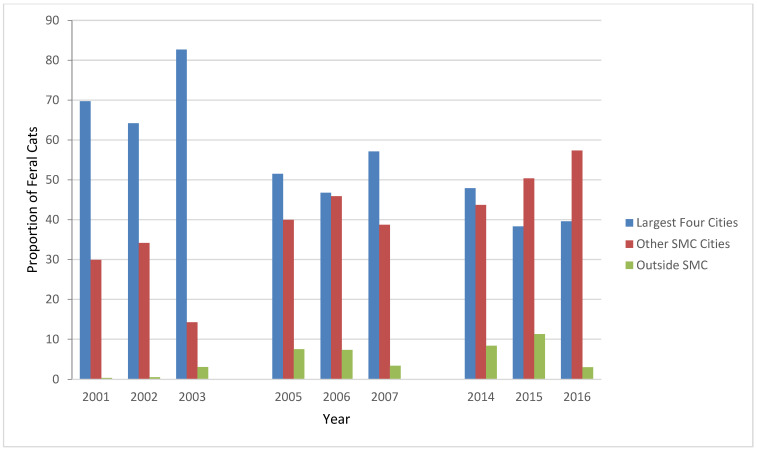
Proportions of feral cats, by source location, presented to the PHS Spay/Neuter Clinic, 2001–2003, 2005–2007, and 2014–2016. SMC—San Mateo County.

During the three study periods, feral cats were presented to the S/N Clinic from the 15 cities and five towns in SMC and from unincorporated areas of the County, San Francisco International Airport (SFO, part of San Francisco though surrounded by SMC), and several cities outside SMC (Table 4). It was not possible to determine whether the cities the caretakers reported were the locations of the feral cats presented to S/N Clinic, or the hometowns of the caregivers themselves. Until 2015, the majority of feral cats presented to the S/N Clinic were from the four SMC cities with the largest human population: San Mateo, South San Francisco, Daly City, and Redwood City (Table 4, Figure 5) [1]. The sources of the feral cats, the four largest SMC cities, other SMC cities, and outside the County, were significantly different in each period (*p* < 0.01).

In the first 3-year period, 30 FeLV-positive cats were associated with nine SMC cities, one FeLV-positive cat from outside SMC, and 101 FIV-positive cats were associated with 17 SMC cities. In the second period, 21 FeLV-positive cats were associated with ten cities and one outside the County. There were 121 FIV-positive cats associated with 17 SMC cities and five with outside cities. In the third period, the two FeLV-positive cats were associated with two cities and none were from outside the County; 38 FIV-positive cats were associated with 16 SMC cities, and seven cats were from outside SMC.

The four male cats in the first study period that were both FeLV- and FIV-positive were from four cities in SMC: San Mateo, South San Francisco, San Bruno, and Burlingame. In the second period, the four male cats were from three cities in SMC (San Mateo, Redwood City, and San Bruno). The two female cats in this second period positive for both retroviruses were from two of the same cities (Redwood City and San Bruno).

There were no significant differences in the overall study period in the proportions of FeLV-positive cats from the four largest cities, compared with the other SMC cities or with the cities outside SMC (Table 5). There was no significant difference in FeLV prevalence in cats from the four largest cities compared with the other cities in SMC and outside the County for the first study period (*p* = 0.07), the second period (*p* = 0.12), or the third (*p* = 1.00). There was no significant difference between FeLV cats by cities for each sex.

There was a significant difference in the proportion of FIV-positive cats from the four largest cities compared with the other cities within SMC during 2001–2003 (*p* < 0.01) (Table 6). There was no significant difference in FIV prevalence in cats from the four largest cities compared with the other cities in SMC and outside the County for the second period (*p* = 0.83) or the third (*p* = 0.44). When evaluated by sex, there were no significant differences between FIV-positive female cats by cities. There was, however, a significant difference in the proportion of male FIV-positive cats from the four largest cities compared with the other cities within SMC in the first 3-year period. Specifically, 43 of 65 (66.15%) FIV-positive male cats were from the four largest cities and 22 (33.85%) from the other SMC cities; none were from outside SMC (*p* < 0.01). In the second and third study periods, no significant differences were found in the proportions of male FIV-positive cats from the four largest cities compared with the other cities inside and outside SMC (*p* = 0.69 and *p* = 0.80, respectively).

### 3.3. Shelter

Between 2001 and 2016, 65,616 live cats were admitted to the Shelter. Of these, 8421 (12.83%) were identified as feral (Table 1, Figure 1). Over this period, the number of live feline admissions decreased significantly from 5781 in 2001 to 2528 in 2016; a significant reduction in admissions was also found in feral admissions (1056 in 2003 to 156 in 2015). The reductions in numbers of live feline admissions (56.27%) and of feral cat admissions (46.38%) were both significant (*p* < 0.01). Few of the feral cats were tested for FeLV or FIV; as such, it would not be meaningful to compare their retroviral prevalence to those of socialized cats in the Shelter tested prior to adoption or to the feral cats presented to the S/N Clinic by feral cat colony caretakers. The sex of the feral cats presented to the Shelter was not recorded.

Cities associated with feral cats presented to the Shelter between 2001 and 2016 showed a similar pattern as observed for the S/N Clinic. In the first 3-year period, the source cities for 21 feral cats were outside SMC and five from SFO, with unknown city data for the remaining 73 cats in the overnight drop-off. The four largest cities in SMC were primary sources of feral cats admitted (60.96%); these were the same four cities from which the majority of feral cats were presented to the S/N Clinic in this period. Between 2005 and 2007, 54.09% of feral cats admitted to the Shelter were from these four cities. The source cities for 20 feral cats were outside SMC and 24 were from SFO, with 180 cats having no city data. During the third period, 50.00% of feral cats admitted to the Shelter were from the four cities. The source cities for 37 feral cats were outside SMC and one was from SFO.

Overall, in the first study period, the four largest cities were the sources of 72.33% of feral cats presented to the S/N Clinic, 83.33% of FeLV-positive feral cats, and 61.39% of FIV-positive feral cats. Correspondingly, for the second study period, the four largest cities were the sources of 55.11% of feral cats, 61.90% of FeLV-positive feral cats, and 60.33% of FIV-positive feral cats. In the third period, these proportions were 45.55%, 50.00%, and 42.10% (Table 7).

## 4. Discussion

The prevalence of FeLV and FIV in feral cats presented to the PHS S/N Clinic differed from prevalence reported at similar clinics in other parts of the country, such as North Carolina and Florida, at the start of this study in 2004; specifically, prevalence was higher for FIV and lower for FeLV [20,21]. While the higher prevalence of FIV among males compared with females was expected, it was unexpected that the trend increased during the second and third study periods. The increasing prevalence of FIV in females in the third period suggests that some retrovirus-positive cats may have been returned to the colonies either without testing or regardless of positive test results, or that new cats entered the colonies. The FeLV prevalence at the S/N Clinic was generally lower than previously reported, with no sex difference in the first period and lower prevalence among females in the second period; no females tested positive in the third period. During the entire study period, there was a 70.03% reduction in total cats and 61.63% in feral cats presented to the S/N Clinic; for the Shelter, the corresponding reductions were 49.82% and 69.08%. Despite these decreases, FIV prevalence remained elevated.

The decrease in Shelter admissions cats (live and specifically feral) suggests that the availability of spay-and-neuter services in SMC has been beneficial in reducing the overall cat population. While other veterinary services are available in SMC, PHS is the largest provider of services to stray and feral animals in the County. It is likely that this trend in decreasing admissions may be attributed in some part to long-term spay and neuter services and public education. This pattern in reduced admission was also noted in the largest neighboring county shelter, San Jose Animal Care & Control (SJACC) [28], where feline admissions declined between 2006 and 2016 from 10,732 to 8489 (*p* < 0.01) [28]. Of these, 10,149 were admitted as stray in 2006; 7962 cats were so classified in 2013 [28]. During these years, a TNR program was initiated by SJACC, which appeared to contribute to decreased overall feline admissions and significantly increased adoptions of socialized stray cats (*p* < 0.01) [28]. This represented a 20.91% and 21.55% reduction in admissions of all cats and feral cats, respectively [28].

Local small animal veterinarians in SMC also accepted vouchers for spay/neuter between 2001 and 2016. The numbers and FeLV/FIV status of feral cats served by private practice veterinarians in SMC is unknown but is expected to be fewer than those presented to the S/N Clinic, as most of these veterinarians serve socialized cats and other animals through the voucher program. Other rescue organizations providing sterilization services for stray cats opened during the study period; however, given the reduction in feline and feral cat admissions to the Shelter, the reduction in S/N Clinic feral cat admissions cannot be explained entirely by the presence of outside services. The S/N Clinic and the Shelter serve different “populations” of feral cats, namely those with dedicated caretakers and those without advocates beyond the Shelter. Thus, the contemporaneous decrease in their numbers suggests that these reductions are an accurate reflection of SMC’s feral cat population trend. While not all feral cats live in colonies or are trapped, this should be nondifferential across groups. Comparisons between the cities associated with cats presented to the S/N Clinic and the Shelter may not be appropriate, since the S/N Clinic cats’ cities likely reflect the addresses of caretakers, while the Shelter cats’ cities may reflect the sites where the cats were found. The similar proportions of Shelter feral cat admissions from the same cities indicate that caretakers’ cities were a reasonable surrogate for their cats’ locations.

The prevalence of FeLV and FIV in specific sites in the United States, Canada, South America, the United Kingdom, Europe, Africa, Australia, New Zealand, and smaller islands has been reported among shelter, stray, and/or identified feral cats [29,30,31,32,33,34,35,36,37,38,39,40,41,42,43,44,45,46,47,48,49,50,51,52,53,54,55,56,57,58,59]. Studies included small numbers of stray cats to large convenience populations at weekend TNR clinics (ranging between 20 and several thousand) with FeLV and FIV prevalence ranging between 0% and 10.4% and between 0% and 36%, respectively [29,30,31,32,33,34,35,36,37,38,39,40,41,42,43,44,45,46,47,48,49,50,51,52,53,54,55,56,57,58,59]. The variety of study designs, including population, numbers, and agencies, make direct comparisons or generalizations about retrovirus prevalence findings difficult. This illustrates the importance of determining the prevalence of these feline retroviruses in the geographic area of interest while evaluating the effectiveness of TNR programs in the given area. Despite these limitations in retrovirus prevalence determination, numerous studies have identified increased FIV prevalence among intact males, increased age, and prior trauma, as was determined in this study [37,38,39,40,41,43,44,45,47,49,50,51,52,53,59].

While the euthanasia policy for retrovirus-positive cats being spayed or neutered using a SMC voucher was concerning to some of the colony caretakers at the start of this study, the veterinarians in the S/N Clinic have indicated that feral cats testing positive for FeLV and/or FIV were always sickly in appearance; that is, they did not euthanize healthy-appearing cats simply due to test results and the voucher stipulation. Thus, euthanasia decisions were not made solely on the results of FeLV/FIV testing, but rather on the combination of test results and clinical presentation, and consideration of the welfare of each cat in free-living conditions. Since only cats presented to the S/N Clinic with the voucher were required to be tested and euthanized if positive for FeLV or FIV, other cats that tested positive may have been returned to their colonies. The results of this study agree with the findings of the PBC study that some FIV-positive cats were returned to colonies in the area served by PHS, rather than euthanized at the time of neutering and testing [15]. During the years of this study, some feral cat colony caretakers who objected to the voucher policy did not use the vouchers to pay for spay/neuter services at the PHS S/N Clinic or used the spay/neuter services of veterinarians who did not test cats for retroviral status. Thus, retroviral-positive feral cats may not have been identified, and/or if they did test positive, they may not have been euthanized, suggesting that FIV-positive cats may have been returned to their colonies without testing or in spite of positive test results.

Concerns regarding retroviral testing of feral cats in high-volume clinics include the cost of tests, time involved in testing in these settings, additional time required to contact feral cat caregivers to discuss disposition for positive cats, and the perceived reduced likelihood of retroviral spread by virus-positive cats once they have been spayed or neutered. Since the S/N Clinic is a brick-and-mortar facility with spay and neuter procedures arranged via appointments, established costs for these procedures and testing, and information provided to caretakers as part of the appointment process, the first three of these arguments are not applicable. The last argument may be the least valid, since returning a virus-positive cat back into the free-roaming cat population re-introduces the potential for disease spread in a group that is unlikely to receive regular or even sporadic veterinary care. The difference between these approaches can be described as driven by facility, time, and money. In the S/N Clinic setting, testing and euthanizing have not been impacted by these considerations. In a volunteer, weekend, high-throughput feral cat spay/neuter clinic, these matter tremendously. Thus, any decision about whether to test and euthanize must take these factors into account.

It must be recognized that simply altering a virus-positive cat does not mean that this cat cannot become a source of infection for cats that have not yet been spayed or neutered, especially in the first weeks following surgery; that is, they may provide a nidus of infection in a colony. The retroviral guidelines of the American Association of Feline Practitioners (AAFP) encourage veterinarians to explain to owners in detail why socialized virus-positive cats should not go outdoors so as to prevent the spread of viruses [16]. Why do we not have this ‘public health’ view with regard to feral cats? It is not a trivial matter to euthanize virus-positive cats based on their test results, but positive cats displaying clinical signs of disease are unlikely to benefit from return to their colonies, either for themselves or in relation to the other cats. For some of these cats, their only contact with veterinary medicine is through the TNR clinic, such that the sequelae of their virus-positive status may not be identified or treated. We must be advocates for the feral cats that test positive, as well as other feral cats these cats will contact. Testing must be performed thoughtfully and each cat’s test result should be one part of a larger evaluation of clinical status.

The AAFP guidelines have recommended keeping retrovirus-positive cats indoors [16,17], which for feral cats, may not be an option unless they have already been socialized. The guidelines noted that cats’ retrovirus status should be known through regular testing to avoid “inadvertent exposure and transmission to uninfected cats” [17]. Cats infected with FeLV “should be confined indoors” so as not to “pose a risk of infection to other cats” and to protect against other infectious agents; cats infected with FIV should be separated from other housecats that are not infected; and cats with either FIV, FeLV, or both should remain indoors to prevent viral spread, and to avoid other infections and stressful environments; and they should receive regular preventive care [17]. These guidelines noted that these testing recommendations need not apply to TNR programs when resources are limited; feral cat testing in TNR programs was considered optional in these cases [17]. The updated retroviral guidelines again note that due to costs, cats in TNR programs should not be tested for FeLV and FIV, yet retrovirus-positive cats should be segregated, a difficult task for most TNR situations [17].

Microchips have been recommended for feral cats as a means to reunite these cats with their caretakers and colonies [60]. This has been optional for feral cats presented to the S/N Clinic. Feral cat caretakers frequently recognize their colonies’ cats on sight. Ear-tipping is used as a sign that a cat has already been spayed or neutered, such that ear-tipped trapped cats can be released before transport for surgery. Other indications of prior sterilization include tattoos (ink used along the incision line). Microchipping has been used more often by SJACC [28], the largest shelter in SCC and by PBC after 2007 [15], but only periodically at PHS.

Some studies have examined the sensitivity and specificity of point-of-care testing for FeLV and FIV [61]. These parameters are characteristics of the test. Test sensitivity is defined as the proportion of truly diseased animals that test positive, while specificity is the proportion of truly disease-free animals that test negative. Higher sensitivity is important to rule out disease and when there is a penalty for missing a disease. Higher specificity is important to confirm disease, when a false positive could harm a patient, and is most helpful if the result is positive. Stated another way, in a screening program, a negative test is helpful when the test has high sensitivity, because there can be greater confidence in ruling out a disease. Another characteristic of testing is positive predictive value (PPV), which is the probability that a test that is positive indicates that the animal has the disease; it is a function of sensitivity, specificity, and prevalence. PPV increases with a higher test specificity and higher disease prevalence. PPV can be improved by only testing based on referrals, or selected groups, or based on specifics of a clinical situation. To make better use of the test, some strategies may be used, such as only testing a population suspected of having disease, rather than all animals. This *artificially* changes the prevalence of disease, and thus improves PPV. For example, suppose that only male cats are tested (thus, half as many cats) and the presumed FIV prevalence among male cats is higher than for females. With increased prevalence, there may be fewer false positives than true positives. Cost savings accrue since the total number of animals tested is reduced; however, there are non-fiscal costs associated with animals that are truly positive for disease that are not tested and re-enter the environment, both for these animals and any with which they come into contact. The decision concerning testing when prevalence is low, or thought to be low, requires a cost–benefit analysis. Without testing, female cats that are positive will not be identified or removed from the population, and may continue to spread disease, albeit passively, through cats that initiate fights with them (FIV) or otherwise have close contact (FeLV). Thus, all tests must be used wisely as just one component of diagnostic or surveillance processes.

Because feral cats are not routinely evaluated by veterinarians following their TNR experience, it can be argued that returning cats that test positive for FeLV or FIV to their communities may be placing a source of infection into the environment. Certainly many community cat caregivers diligently follow the health of the cats for whom they care; however, this surveillance is passive by design. Thus, the potential for transmission of infection among cats in a community may not be completely eliminated by the TNR experience alone. Humane education and outreach is required to address FIV prevalence in SMC, and it is important that disagreements about managing community cat colonies on the basis of health status be resolved to keep outdoor cats healthy. Enhanced communications among all who work with community cats is required to protect cats from disease. While the more recent AAFP Guidelines regarding FeLV- and FIV-positive cats pertain to owned cats, applying such guidelines to community cats has not be recommended [17], adding to the discussion that veterinarians should have with community cat caretakers and owners whose cats venture outdoors. The increase in FIV prevalence among female cats in the third period of this study suggests that active surveillance should continue in SMC.

Limitations of this study include incomplete information regarding the feral cats presented to the S/N Clinic, though the proportion of such information was small. Missing or incomplete data in the S/N Clinic data may have contributed to misallocation; however, the records of 4.11% of feral cats were missing in the first 3-year period, with even fewer missing data in the latter two periods. Because the Shelter did not record sex or FeLV/FIV status on admitted feral cats, no disease prevalence comparison was possible with the cats presented to the S/N Clinic in managed feral cat colonies. While the cities were identified with feral cats admitted to the Shelter, the cities listed for cats presented to the S/N Clinic likely represented the caretakers’ residences, rather than the cities of the feral cat colonies they manage; however, education campaigns for spaying and neutering should be directed to populations with feral cats and to feral cat colony caretakers, regardless of where the cats themselves may be.

## 5. Conclusions

Over the three 3-year study periods, the prevalence of FIV increased from 5.52% to 6.41% (*p* = 0.29) and the prevalence of FeLV decreased significantly from 1.73% to 0.29% (*p* < 0.02). The prevalence of these retroviruses was different from those of other populations in earlier U.S. studies and later worldwide studies. For each period of the study, there were significantly more FIV-positive males than females (*p* < 0.01). Despite a 61.63% decrease in feral cat admissions to the S/N Clinic in the third period, the prevalence of FIV for males remained similar across the years and increased for females. This increasing trend suggests the need for continued testing and surveillance of FIV among free-living community cats in SMC. Enhanced communications among all who work with community cats is required to protect cats from disease. The identification of feral cat population centers with increased retrovirus prevalence in the largest SMC cities can be used to focus attention on surveillance activities and humane education for caretakers in these areas. The impression from early studies that feline retroviral prevalence is low among feral cats in the U.S. has made testing a source of financial and emotional contention. Because some cat caretakers in the area have ceased using the SMC voucher services at the PHS S/N Clinic due to the testing policy, it is important that disagreements about managing community cat colonies on the basis of health status be resolved to keep outdoor cats healthy.

## Figures and Tables

**Table 1:** Numbers and proportions of feral cats admitted to the PHS Shelter and the Spay/Neuter Clinic, 2001–2016.

	Spay/Neuter Clinic			Shelter		
Year	Feral Cats	Total Cats	%	Feral Cats	Total Cats	%
2001	568	3248	17.49	414	5781	7.16
2002	726	3860	18.81	681	5029	13.54
2003	554	3881	14.27	1056	5354	19.72
2004				901	4670	19.29
2005	598	3117	19.19	732	4615	15.86
2006	667	3330	20.03	672	4403	15.26
2007	653	3340	19.55	582	4350	13.38
2008				450	4076	11.04
2009				476	4323	11.01
2010				439	4208	10.43
2011				510	3850	13.25
2012				460	3519	13.07
2013				383	3327	11.51
2014	238	1076	22.12	287	2704	10.61
2015	274	1129	24.27	156	2879	5.42
2016	197	1088	18.11	222	2528	8.78

**Table 2:** Feline retroviral prevalence among feral cats presented to the PHS Spay/Neuter Clinic in the years 2001–2003, 2005–2007, and 2014–2016 *.

Year	N	FeLV+ (%)	FIV+ (%)
2001	568	9 (1.58)	34 (5.99)
2002	726	8 (1.10)	39 (5.37)
2003	554	15 (2.71	29 (5.23)
		32 (1.73)	102 (5.52)
2005	598	8 (1.42)	45 (7.74)
2006	667	8 (1.23)	37 (5.68)
2007	653	6 (0.94)	44 (6.89)
		22 (1.19)	126 (6.73)
2014	238	1 (0.43)	15 (6.36)
2015	274	0 (0.00)	19 (6.99)
2016	197	1 (0.52)	11 (5.67)
		2 (0.29)	45 (6.41)

* Totals for viral status do not sum to total numbers due to incomplete information; N = number of cats; FeLV+—feline leukemia virus-positive; FIV+—feline immunodeficiency virus-positive.

**Table 3:** Feline retroviral prevalence, stratified by sex, among feral cats presented to the PHS Spay/Neuter Clinic in the years 2001–2003, 2005–2007, and 2014–2016 *.

				FeLV+		FIV+	
Year	N	Male	Female	Male	Female	Male ^a^	Female
2001	568	249	293	4 (1.61)	4 (1.37)	20 (8.03)	5 (1.71) ^b^
2002	726	321	361	2 (0.62)	4 (1.11)	25 (7.79)	7 (1.94) ^b^
2003	554	244	304	8 (3.28)	6 (1.97)	20 (8.20)	8 (2.63) ^b^
		814	958	14 (1.72)	14 (1.46)	65 (7.99)	20 (2.09) ^b^
2005	598	240	356	5 (2.24)	3 (0.88)	33 (14.29)	10 (2.87) ^b^
2006	667	295	372	7 (2.46)	1 (0.27) ^c^	31 (10.80)	6 (1.65) ^b^
2007	653	281	370	2 (0.72)	4 (1.12)	38 (13.62)	4 (1.12) ^b^
		816	1098	14 (1.79)	8 (0.75) ^e^	102 (12.80)	20 (1.87) ^b^
2014	238	110	128	1 (0.92)	0 (0.00)	13 (11.82)	2 (1.59) ^b^
2015	274	109	163	0 (0.00)	0 (0.00)	12 (11.01)	6 (3.73) ^d^
2016	197	99	96	1 (1.04)	0 (0.00)	10 (10.31)	1 (1.05) ^b^
		318	387	2 (0.64)	0 (0.00)	35 (11.08)	9 (2.36) ^b^

* Totals for sex and viral status do not sum to total numbers due to incomplete information; N = number of cats; FeLV+—feline leukemia virus– positive; FIV+—feline immunodeficiency virus-positive. Note: ^a^—p_MH_ < 0.04—increasing trend for males across years; *p* = 0.02 across periods; ^b^—*p* < 0.01; ^c^—*p* = 0.02; ^d^—*p* < 0.02; ^e^—*p* = 0.04.

**Table 4:** Sources of feral cats presented to PHS S/N Clinic, 2001–2003, 2005–2007, and 2014–2016 *.

Year	N	Largest Four Cities (%)	Other SMC (%)	Outside SMC (%)
2001	568	396 (69.72)	170 (29.93)	2 (0.35)
2002	726	466 (64.90)	248 (34.54)	4 (0.56)
2003	554	458 (82.67)	79 (14.26)	17 (3.07)
		1320 (71.74) ^a^	497 (27.01)	23 (1.25)
2005	598	308 (52.03)	239 (40.37)	45 (7.60)
2006	667	312 (46.78)	306 (45.88)	49 (7.35)
2007	653	373 (57.56)	253 (39.04)	22 (3.40)
		993 (52.07) ^a^	798 (41.85)	116 (6.08)
2014	238	114 (46.15)	113 (45.75)	20 (8.10)
2015	274	105 (39.62)	129 (48.68)	31 (11.70)
2016	197	78 (39.59)	113 (57.36)	6 (3.05)
		297 (41.89) ^a^	355 (50.07)	57 (8.04)

* Totals for source location do not sum to total numbers due to incomplete information. N—number of cats; SMC—San Mateo County. Note: ^a^—*p* < 0.01.

**Table 5:** Feline leukemia virus positive prevalence, stratified by source location within San Mateo County, among feral cats presented to the PHS Spay/Neuter Clinic in the years 2001–2003, 2005–2007, and 2014–2016 *.

Year	N	Largest Four Cities (%)	Other SMC (%)	Outside SMC (%)
2001	9	5 (55.55)	4 (44.44)	0 (0.00)
2002	7	7 (100.00)	0 (0.00)	0 (0.00)
2003	15	13 (86.67)	1 (6.67)	1 (6.67)
		*25 (80.64)*	*5 (16.13)*	*1 (3.23)*
2005	8	7 (87.50)	1 (12.50)	0 (0.00)
2006	8	2 (25.00)	5 (62.50)	1 (12.50)
2007	6	4 (66.67)	2 (33.33)	0 (0.00)
		13 (59.09)	8 (36.36)	1 (4.54)
2014	1	1 (100.00)	0 (0.00)	0 (0.00)
2015	0	0 (0.00)	0 (0.00)	0 (0.00)
2016	1	0 (0.00)	1 (100.00)	0 (0.00)
		1 (50.00)	1 (50.00)	0 (0.00)

* Totals for source location do not sum to total numbers due to incomplete information. N—number of cats; SMC—San Mateo County.

**Table 6:** Feline immunodeficiency virus positive prevalence, stratified by source location within San Mateo County, among feral cats presented to the PHS Spay/Neuter Clinic in the years 2001–2003, 2005–2007, and 2014–2016 *.

Year	N	Largest Four Cities (%)	Other SMC (%)	Outside SMC (%)
2001	34	18 (52.94)	16 (47.06)	0 (0.00)
2002	38	18 (47.37)	20 (52.63)	0 (0.00)
2003	29	26 (89.66)	3 (10.34)	0 (0.00)
		62 (61.39) ^a^	39 (38.61)	0 (0.00)
2005	45	26 (57.78)	17 (37.78)	2 (4.44)
2006	37	19 (51.35)	16 (43.24)	2 (5.40)
2007	44	28 (63.64)	15 (34.09)	1 (2.27)
		73 (57.94)	48 (38.09)	5 (3.97)
2014	15	4 (26.67)	7 (46.67)	4 (26.67)
2015	19	9 (47.37)	8 (42.10)	2 (10.53)
2016	11	3 (27.27)	7 (63.64)	1 (9.09)
		16 (35.56)	22 (48.89)	7 (15.56)

* Totals for source location do not sum to total numbers due to incomplete information; Note: ^a^—*p* < 0.01. N—number of cats; SMC—San Mateo County.

**Table 7:** Proportions of feral cats presented to PHS Shelter and S/N Clinic in the three periods from the four largest cities in San Mateo County *.

	2001–2003	2005–2007	2014–2016
Feral cats admitted to Shelter	60.96	54.09	50.00
Feral cats presented to S/N Clinic	72.33	55.11	45.55
FeLV+ of tested S/N Clinic feral cats	83.33	61.90	50.00
FIV+ of tested S/N Clinic feral cats	61.39	60.33	42.10

* Based on cats from San Mateo County only. S/N—Spay/Neuter; FeLV+—feline leukemia virus-positive; FIV+—feline immunodeficiency virus-positive.

## Data Availability

Not applicable.
